# A Pilot Study of Gender Differences in Sexual Arousal of Patients With OCD: The Moderator Roles of Attachment and Contamination Symptoms

**DOI:** 10.3389/fpsyt.2020.609989

**Published:** 2021-02-10

**Authors:** Davide Dèttore, Nicole Loren Angelo, Donatella Marazziti, Federico Mucci, Davide Prestia, Andrea Pozza

**Affiliations:** ^1^Department of Health Sciences, University of Florence, Florence, Italy; ^2^School of Psychology, University of Florence, Florence, Italy; ^3^Department of Clinical and Experimental Medicine, Section of Psychiatry, University of Pisa, Pisa, Italy; ^4^UniCamillus - Saint Camillus International University of Health Sciences, Rome, Italy; ^5^Department of Biotechnology, Chemistry and Pharmacy, University of Siena, Siena, Italy; ^6^Section of Psychiatry, Department of Neuroscience, Rehabilitation, Ophthalmology, Genetics and Infant-Maternal Science, University of Genoa, Genoa, Italy; ^7^IRCCS Ospedale Policlinico San Martino, Genoa, Italy; ^8^Department of Medical Sciences, Surgery and Neurosciences, University of Siena, Siena, Italy

**Keywords:** obsessive-compulsive disorder, sexual well-being, quality of life, gender, attachment, sexual pleasure, contamination symptoms

## Abstract

Sexual arousal is often impaired in patients with obsessive–compulsive disorder (OCD). However, little is known about the factors related to this impairment: no study focused on the role of gender-based effects of attachment styles and contamination symptoms. The Dual Control Model assumes three processes driving sexual arousal: sexual excitation (SE), sexual inhibition (SI) due to threat of performance failure, and SI due to threat of performance consequences (e.g., getting contaminated with sexually transmitted diseases). In a group of OCD patients, we hypothesized that (a) women report lower SE and higher SI than men; (b) patients with insecure (both anxious and avoidant) attachment styles show lower SE and higher SI; (c) attachment styles moderate the relation between gender and sexual arousal (respectively, for women, higher attachment anxiety, and for men higher attachment avoidance were related to impaired sexual arousal (higher SE and SI) controlling for OCD severity); and (d) contamination symptoms moderate the relation between gender and sexual impairment (women with contamination symptoms show impaired sexual arousal). Seventy-two OCD patients (37.50% women) completed the Obsessive–Compulsive Inventory-Revised, Attachment Styles Questionnaire and Sexual Inhibition/Sexual Excitation Scales. In contrast with our hypotheses, women reported higher SE and lower SI due to threat of performance consequences than men. Patients with higher attachment avoidance (discomfort with intimacy) but also confidence in self and others had higher SE. Women with attachment avoidance (i.e., discomfort with intimacy) had lower SE, while women with attachment anxiety (i.e., preoccupations with relationships) had higher SI due to negative performance consequences. Women with contamination symptoms had higher SI due to performance failure but lower SI due to performance consequences. The present preliminary findings suggest that sexual arousal impairment should be evaluated during the assessment of OCD patients, and gender-based effects of attachment styles and contamination symptoms should be considered during personalized treatment planning.

## Introduction

### Sexual Arousal in Obsessive–Compulsive Disorder: The Role of Gender According to the Dual Control Model

Obsessive–compulsive disorder (OCD) is a psychiatric condition characterized by obsessions and compulsions that cause significant distress and disability in various aspects of quality of life (1, 2). Therefore, it is not surprising that sexual functioning, significantly contributing to quality of life, may be altered in this clinical population, since this type of patients more frequently present sexual dysfunctions such as less sexual desire and arousal, and orgasmic difficulties than people of the general population (3–5). Some data showed that patients with OCD report more frequent and more intense sexual dysfunctions even than other clinical groups such as patients with anxiety disorders (6).

Indeed, impaired sexual functioning in OCD patients may be influenced by serotonergic antidepressant medication, commonly prescribed at high dosages as the first-line psychopharmacological intervention, which can induce some sexual dysfunctions both in men and women (7). However, not all data supported this evidence, and some papers suggested that OCD patients may have sexual dysfunctions independently of serotonergic medications (8). Data from meta-analyses and from empirical studies indicated that the female gender would be related to a worse intimate and interpersonal quality of life (9) and more frequent sexual dysfunctions than the male gender (10, 11).

The Dual Control Model (DCM) (12) highlights the psychological processes related to impairment in sexual functioning. In agreement with this theoretical framework, a healthy sexual arousal relies on a balanced relation between sexual excitation (SE) and sexual inhibition (SI), that is to say, high and low levels of excitation and inhibition, respectively. Three psychological processes driving sexual arousal are hypothesized: (a) the level of SE, (b) the level of SI due to threat of performance failure, and (c) the level of SI due to threat of performance consequences (e.g., getting contaminated with sexually transmitted diseases). Similar to a gas pedal, SE influences how easily the individual becomes aroused by internal (e.g., fantasies) or external cues (e.g., a sexually attractive partner). SI, conceptualized as a brake pedal, reduces sexual arousal, and discourages sexual behavior when the context is inappropriate, or the pursuit of sexual activities poses a threat to the person (13). According to this model (12, 13), too low or too high levels of SE and/or SI may be associated with an unbalanced sexual response, thus in an impairment in sexual life.

The DCM considers that sexual functioning is influenced by individual factors, one of which is gender (12). High levels of SI due to threat of performance failure were indicative of erectile dysfunction in community samples of men, even though they tended to manifest high levels of SE, while women were less aroused and more sexually inhibited (12). Recent studies in community samples showed that women are more likely to experience a lower and a higher propensity toward, respectively, excitation and inhibition than men (14–21). While the DCM has been extensively used in studying the individual features associated with SE and SI processes in community samples, little is known about the individual features associated with sexual arousal processes in OCD patients based on this theoretical framework.

### The Potential Role of Attachment Styles in Sexual Arousal of Obsessive–compulsive Disorder Patients

Attachment style is the emotional bond developed between infant and caregiver ensuring safety and protection (22, 23). As stated by Hazan and Shaver (24), three following specific patterns would be typical of adult attachment: secure, avoidant, and anxious/ambivalent. On the basis of this model, Bartholomew and Horowitz (25) identified four typical adult attachment styles: secure, preoccupied, fearful-avoidant, and dismissive-avoidant. In sexual relationships, adults manifest the previously developed attachment needs, directing them toward romantic partners and resulting in a specific sexual behavior (26).

Feeney et al. (27) proposed a different theoretical model hypothesizing that attachment is not a categorical construct (i.e., different styles may not be mutually exclusive in the same person) and proposed a dimensional model including five attachment styles: discomfort with closeness, relationships as secondary (e.g., to achievement), need for approval, preoccupation with relationships, and confidence (in self and others). These styles can be understood using the concepts of avoidance and anxiety: discomfort with closeness and relationships as secondary reflect attachment avoidance; need for approval, preoccupation with relationships, and low confidence reflect attachment anxiety (27). The authors developed a self-report measure, the Attachment Style Questionnaire (27), which assesses the five attachment styles as they were introduced by the model.

Generally, secure individuals engage in healthy sexual relationships (22, 26–29). In individuals with attachment anxiety, constant fear of separation can lead to SI (30, 31). In individuals with attachment avoidance, SI may be a consequence of their tendency to maintain sex distinct from the emotional aspects of the relationship (32–37). Research investigating gender differences in attachment styles demonstrated that women report higher levels of attachment anxiety, while men report higher levels of attachment avoidance (35–39).

Attachment style is another aspect that might influence behaviors in intimate relationships of OCD patients since in this clinical population attachment insecurities can often be observed (40–43). Insecure attachment, specifically anxious and avoidant, is typically associated with OCD (40–47). According to a recent meta-analysis, both attachment anxiety and attachment avoidance were associated with OCD (48).

### Contamination Symptoms and Sexual Arousal of Obsessive–compulsive Disorder Patients

Another clinical feature that might moderate the relation between OCD and sexual impairment is the type of obsessions and compulsions. Overall, OCD is a heterogeneous condition that can include different symptom dimensions (49–51). Previous evidence showed that contamination symptoms represent one of the dimensions most strongly related to impaired quality of life (52–54). In a first study on women, none of the symptom dimensions including contamination, moral (aggressive, sexual, and religious), somatic, and symmetry obsessions were found to be related to sexual pleasure and functioning (4). Contamination symptoms might have a severe impact on sexual life specifically, since they typically involve the fear of body contact and the fear of getting sexually transmitted diseases and genital fluids (55). The effect of contamination symptoms on sexual life might vary across gender. Indeed, the majority of studies including recent systematic reviews show that contamination symptoms are generally more frequent among women than men (56–58).

### Rationale and Hypotheses

Little is known about the factors related to sexual arousal impairment among OCD patients: specifically, no study focused on the role of gender. Therefore, the present study aimed to explore the role of gender on sexual arousal processes in patients with OCD (i.e., propensity for SE and SI according to the DCM) and to investigate whether attachment styles and contamination symptoms moderate the relationship between gender and propensity for SE and SI. Based on the literature data about gender differences in attachment styles, contamination symptoms and sexual arousal processes [e.g., (35–39, 55–58)], we hypothesized that (a) female patients report lower SE and higher SI than male ones; (b) OCD patients with more anxious and avoidant attachment styles show lower SE and higher SI; (c) attachment styles moderate the relation between gender and sexual response after controlling for general OCD severity, i.e., respectively, for female patients higher attachment anxiety and for male patients, higher attachment avoidance are related to impaired sexual response (lower SE and higher SI); and (d) contamination symptoms moderate the relation between gender and sexual response, i.e., for female patients, the presence of contamination symptoms is related to impaired sexual response (lower SE and higher SI).

## Methods

### Participants

Inclusion criteria were (i) primary current diagnosis of OCD established by a psychiatrist or psychologist through the Structured Clinical Interview for DSM-IV-TR Axis I Disorders (SCID-I) (59), (ii) age between 18 and 65 years, and (iii) provision of signed informed consent. Exclusion criteria were (i) psychotic disorders, (ii) bipolar disorders, (iii) mental retardation, (iv) neurological disorders, (v) active suicidal ideation, and (vi) drug dependence/abuse. The use of serotonergic medications was not considered an exclusion criterion, since it is commonly used as a first-line treatment in OCD patients (60, 61). All participants were recruited through mental health specialists in public or private centers, and their diagnosis of OCD was made through the SCID-I, and it was confirmed through the Yale-Brown Obsessive–Compulsive Scale (Y-BOCS) (62).

Participation was voluntary and uncompensated. All the subjects were required to provide written informed consent to participate after receiving a full description of the aims and having the possibility to withdraw their consent at any time, without any consequences for their treatment. Materials containing personal information were kept on electronic supports protected by passwords. The research was carried out according to the Helsinki Declaration and approved by the institutional ethics committee.

### Measures

#### Obsessive–Compulsive Inventory-Revised

The Obsessive–Compulsive Inventory-Revised (OCI-R) (63) consists of 18 items divided into six subscales: *Checking, Washing, Obsessing, Mental Neutralizing, Ordering*, and *Hoarding*. Each item is evaluated on a 5-point Likert scale (0 = Not at all, 4 = Extremely). The Italian version presented acceptable internal consistency (α > 0.70), and good test–retest reliability (Pearson's *r* > 0.70) (64). In the present study, internal consistency was good for all the subscales (range of Cronbach's alpha = 0.83–0.88). The presence of contamination symptoms was assessed by a score on the OCI-R Contamination/Washing subscale higher than the score equal to or higher than the 95th percentile in the normal distribution of the Italian validation of the scale (64) and confirmed through the Y-BOCS.

#### Attachment Style Questionnaire

The Attachment Style Questionnaire (ASQ) (27) includes 40 items measuring five attachment styles based on the models proposed by Hazan and Shaver (24) and Bartholomew and Horowitz (25): *Confidence, Discomfort with Closeness, Relationships as Secondary, Need for Approval*, and *Preoccupation with Relationships*. Each item is evaluated on a 6-point Likert scale (1 = Strongly disagree, 6 = Strongly agree). The Italian version showed an acceptable internal consistency (0.67 < α < 0.74) (65). In the present study, internal consistency was acceptable to good (range of Cronbach's alphas = 0.79–0.85).

#### Sexual Inhibition/Sexual Excitation Scales

The Sexual Inhibition/Sexual Excitation Scales (SIS/SES) (66) was developed to assess individual differences in the sexual response. The SIS/SES includes 45 items, divided into three factors: Sexual Excitation (SES; example item: “When I start fantasizing about sex, I quickly become sexually aroused”); Sexual Inhibition Due to Threat of Performance Failure (SIS1; example item: “Once I have an erection, I want to start intercourse right away before I lose my erection/Once I am sexually aroused, I want to start intercourse right away before I lose my arousal”), and Sexual Inhibition Due to Threat of Performance Consequences (SIS2; “If I realize there is a risk of catching a sexually transmitted, I am unlikely to stay sexually aroused”). These three scales are evaluated on a 4-point Likert scale (1 = Strongly Agree, 4 = Strongly Disagree), where lower scores indicate higher SE and SI (66). The Italian version presented acceptable to good psychometric properties (0.69 < α < 0.89; Pearson's *r* > 0.60) (67). In the present study, internal consistency was acceptable to good for all the scales (range of Cronbach's alphas = 0.76–0.82).

### Statistical Analyses

Independent-group Student's *t-*tests were computed to investigate between-group differences regarding the intensity of OCD-related symptoms (OCI-R scores), attachment styles (ASQ scores), and levels of SE and SI (SIS/SES scores) as a function of gender. The Chi-squared test was carried out to explore the association between gender and the presence of contamination symptoms detected by a score on the OCI-R Contamination/Washing subscale higher than the score equal to or higher than the 95th percentile in the normal distribution of the Italian validation of the scale (64), later confirmed through the Y-BOCS administration.

Subsequently, two separate sets of three analyses of generalized linear models were carried out. The first set of three generalized linear models aimed to investigate the moderator role of attachment styles in the relation between gender and sexual arousal impairment controlling for general OCD severity (hypotheses A–C). Thus, in these three models, we included the predictive effects of gender, general intensity of OCD symptoms (OCI-R total scores), attachment styles (ASQ scores), and gender × ASQ scores interaction effects on SIS/SES scores. The second set of three generalized linear models aimed to explore the additional moderator effects of contamination symptoms in the relation between gender and sexual arousal impairment (hypothesis D). The statistical significance was set at *p* < 0.05. The analyses were conducted through the software SPSS version 23.00.

## Results

### Sociodemographic and Clinical Characteristics

Seventy-two patients with OCD (mean age ±*SD*: 34.50 ± 10.39) were included in the study. Twenty-seven were women (37.50%) and 45 men (62.50%). Twenty-seven patients (37.50%) reported contamination symptoms. Sociodemographic and clinical characteristics are shown in Table 1.

**Table 1:** Socio-demographic and clinical characteristics (*n* = 72).

	**M (*SD*; range)/*n* (%)**
Age (years)	34.50 (10.39; 18–58)
**GENDER**	
Female	27 (37.50)
Male	45 (62.50)
**MARITAL STATUS**	
Single	55 (76.40)
Married	15 (20.80)
Divorced	2 (2.80)
**EDUCATION LEVEL**	
Elementary school	3 (4.20)
Middle school	5 (6.90)
High school	35 (48.60)
Degree	24 (33.30)
Post-graduate education	4 (5.60)
**EMPLOYMENT STATUS**	
Undergraduate	1 (1.40)
Employed	16 (22.20)
Unemployed	44 (61.10)
Other	9 (12.50)
Age at OCD onset (years)	21.36 (8.90; 6–53)
Concurrent antidepressant (serotonergic) medication	32 (44.40)

### Moderator Role of Attachment Styles in the Relation Between Gender and SE/SI (Hypotheses A–C)

A first series of comparisons performed by Student's *t*-tests between men and women showed no significant differences across gender on the SIS/SES scores, OCI-R Total, and ASQ scores [range of *t*_(70)_ = −1.87–1.40, *p* = 0.16–0.94].

The results of the non-parametric tests suggested an association between gender and contamination symptoms: the number of women with contamination symptoms was significantly higher than the number of men [χ^2^_(1)_ = 4.50, *p* = 0.034].

Subsequently, a first set of generalized linear models has been carried out to test hypotheses A–C. The results are displayed in Tables 2–4. The model for SE as outcome is depicted in Table 2. On the one hand, higher OCD severity measured by the OCI-R total scores was associated with lower SE (β = 0.012, *p* = 0.004). On the other hand, women reported higher SE than men (β = −3.240, *p* = 0.022). In addition, patients with higher scores on ASQ *Confidence* (β = −0.030, *p* = 0.044) and ASQ *Discomfort with Closeness* (β = −0.030, *p* = 0.024) reported higher SE.

**Table 2:** Generalized linear model of SES scores on gender, OCI-R, and ASQ scores (*n* = 72).

**Outcome: SES**	***B***	***SE***	**95% CI**				
			**Lower**	**Upper**	**Wald's χ^2^**	***df***	***p*-value**
Intercept	4.920	0.8323	3.289	6.552	34.954	1	0.000
Gender	−3.240	1.4113	−6.006	−0.473	5.269	1	0.022
Age	−0.001	0.0058	−0.012	0.011	0.012	1	0.913
OCI-R total	0.012	0.0043	0.004	0.021	8.424	1	0.004
ASQ confidence	−0.030	0.0152	−0.060	−0.001	4.040	1	0.044
ASQ discomfort with closeness	−0.030	0.0134	−0.056	−0.004	5.085	1	0.024
ASQ relationships as secondary	−0.008	0.0134	−0.034	0.018	0.352	1	0.553
ASQ need for approval	0.007	0.0138	−0.021	0.034	0.224	1	0.636
ASQ preoccupation with relationships	−0.021	0.0133	−0.047	0.005	2.506	1	0.113
Gender * ASQ confidence	0.057	0.0249	0.008	0.106	5.288	1	0.021
Gender * ASQ discomfort with closeness	0.051	0.0210	0.010	0.092	5.915	1	0.015
Gender * ASQ need for approval	0.006	0.0202	−0.033	0.046	0.094	1	0.759
Gender * ASQ preoccupation with relationships	0.013	0.0292	−0.045	0.070	0.185	1	0.667
Gender * ASQ relationships as secondary	−0.024	0.0255	−0.074	0.026	0.899	1	0.343

*SE, Standard error; SES, Sexual Excitation Scale; CI, Confidence Interval; OCI-R, Obsessive Compulsive Inventory-Revised; ASQ, Attachment Style Questionnaire*.

There was an interaction effect between gender and ASQ *Confidence* scores and between gender and ASQ *Discomfort with Closeness* scores: women with higher ASQ *Confidence* scores (β = 0.057, *p* = 0.021) and those with higher ASQ *Discomfort with Closeness* scores (β = 0.051, *p* = 0.015) reported lower SE.

None of the predictors examined, i.e., gender, OCD severity, and attachment styles were significantly related to SI due to threat of performance failure as measured by the SIS1 scores (see Table 3 for the model of SI due to threat of performance failure as outcome).

**Table 3:** Generalized linear model of SIS1 scores on gender, OCI-R, and ASQ scores (*n* = 72).

**Outcome: SIS1**	**β**	***SE***	**95% CI**				
			**Lower**	**Upper**	**Wald's *χ^2^***	***df***	***p*-value**
Intercept	2.205	0.9192	0.404	4.007	5.757	1	0.016
Gender	1.692	1.5588	−1.363	4.747	1.178	1	0.278
Age	0.007	0.0064	−0.006	0.019	1.181	1	0.277
OCI-R	−0.007	0.0047	−0.016	0.003	1.933	1	0.164
OCI-R total	0.012	0.0167	−0.021	0.045	0.499	1	0.480
ASQ confidence	−0.015	0.0148	−0.044	0.014	1.057	1	0.304
ASQ discomfort with closeness	−0.009	0.0148	−0.038	0.020	0.393	1	0.531
ASQ relationships as secondary	−0.011	0.0152	−0.041	0.019	0.525	1	0.469
ASQ need for approval	0.026	0.0147	−0.003	0.055	3.119	1	0.077
ASQ preoccupation with relationships	−0.025	0.0276	−0.079	0.029	0.837	1	0.360
Gender * ASQ confidence	0.033	0.0232	−0.013	0.078	1.969	1	0.161
Gender * ASQ discomfort with closeness	−0.011	0.0223	−0.054	0.033	0.238	1	0.625
Gender * ASQ need for approval	−0.042	0.0322	−0.106	0.021	1.737	1	0.187
Gender * ASQ preoccupation with relationships	−0.034	0.0282	−0.089	0.021	1.463	1	0.226

*SE, Standard error; SIS1, Sexual Inhibition Type 1 Scale; CI, Confidence Interval; OCI-R, Obsessive Compulsive Inventory-Revised; ASQ, Attachment Style Questionnaire*.

The model of SI due to threat of performance consequence as outcome is presented in Table 4. Higher ASQ *Confidence* (β = 0.046, *p* = 0.003) and ASQ *Need for Approval* scores (β = 0.051, *p* = 0.000) were associated with lower SI due to threat of performance consequences. In addition, an interaction effect was noted between gender and ASQ *Confidence* scores, and between gender and ASQ *Need for Approval* scores. Indeed, OCD female patients with higher ASQ *Confidence* scores (β = −0.070, *p* = 0.005) and higher ASQ *Preoccupations with Relationships* scores (β = −0.108, *p* = 0.000) resulted more threatened by potentially negative performance consequences.

**Table 4:** Generalized linear model of SIS2 scores on gender, OCI-R, and ASQ scores (*n* = 72).

**Outcome: SIS2**	**β**	***SE***	**95% CI**				
			**Lower**	**Upper**	**Wald's *χ^2^***	***df***	***p*-value**
Intercept	0.606	0.8396	−1.040	2.251	0.520	1	0.471
Gender	2.652	1.4238	−0.138	5.443	3.470	1	0.062
Age	0.007	0.0058	−0.005	0.018	1.304	1	0.253
OCI-R total	−0.009	0.0043	−0.017	−5.808*E*−5	3.894	1	0.048
ASQ confidence	0.046	0.0153	0.016	0.076	9.074	1	0.003
ASQ discomfort with closeness	−0.022	0.0135	−0.048	0.005	2.577	1	0.108
ASQ relationships as secondary	0.002	0.0135	−0.025	0.028	0.017	1	0.897
ASQ need for approval	0.051	0.0139	0.024	0.078	13.493	1	0.000
ASQ preoccupation with relationships	−0.008	0.0134	−0.035	0.018	0.387	1	0.534
Gender * ASQ confidence	−0.070	0.0252	−0.120	−0.021	7.798	1	0.005
Gender * ASQ discomfort with closeness	0.013	0.0212	−0.029	0.054	0.359	1	0.549
Gender * ASQ relationships as secondary	−0.008	0.0203	−0.048	0.032	0.155	1	0.693
Gender * ASQ need for approval	−0.108	0.0294	−0.166	−0.050	13.494	1	0.000
Gender * ASQ preoccupation with relationships	0.050	0.0257	0.000	0.101	3.817	1	0.051

*SE, Standard error; SIS2, Sexual Inhibition Type 2 Scale; CI, Confidence Interval; OCI-R, Obsessive Compulsive Inventory-Revised; ASQ, Attachment Style Questionnaire*.

### Moderator Role of Contamination Symptoms in the Relation Between Gender and SE/SI (Hypothesis D)

When the effects of contamination symptoms were added in the generalized linear model, the results (Table 5) showed again that women had higher SE than men (β = −3.336, *p* = 0.023). Patients with higher ASQ *Discomfort with Closeness* scores had higher SE (β = −0.032, *p* = 0.025). In addition, there was an interaction effect between gender and ASQ *Confidence* scores and between gender and ASQ *Discomfort with Closeness* scores: women with higher confidence (β = −0.057, *p* = 0.021) and those with higher discomfort with intimacy (β = −0.051, *p* = 0.015) reported higher SE. No effect was found for contamination symptoms or their interaction with gender. For SI due to threat of performance failure (Table 6), there was only an interaction effect between gender and contamination symptoms (β = −0.060, *p* = 0.30), suggesting that women with contamination symptoms had higher SI due to threat of performance failure. The other predictors were not significant in the model.

**Table 5:** Generalized linear model of SES scores on gender, contamination symptoms and ASQ scores (*n* = 72).

**Outcome: SES**	**β**	***SE***	**95% CI**				
			**Lower limit**	**Upper limit**	**Wald *χ^2^***	***df***	***p*-value**
Intercept	4.880	0.8612	3.192	6.567	32.104	1	0.000
Gender	−3.336	1.4633	−6.204	−0.468	5.198	1	0.023
Age	0.000	0.0060	−0.011	0.012	0.004	1	0.948
Contamination symptoms	0.333	0.1765	−0.013	0.679	3.553	1	0.059
ASQ confidence	−0.026	0.0153	−0.055	0.004	2.796	1	0.095
ASQ discomfort with closeness	−0.032	0.0141	−0.059	−0.004	5.052	1	0.025
ASQ relationships as secondary	−0.006	0.0137	−0.033	0.020	0.222	1	0.637
ASQ need for approval	0.016	0.0147	−0.013	0.045	1.233	1	0.267
ASQ preoccupation with relationships	−0.025	0.0148	−0.054	0.004	2.779	1	0.095
Gender * ASQ confidence	0.057	0.0254	0.008	0.107	5.090	1	0.024
Gender * ASQ discomfort with closeness	0.051	0.0236	0.005	0.097	4.685	1	0.030
Gender * ASQ need for approval	0.012	0.0207	−0.029	0.052	0.324	1	0.569
Gender * ASQ preoccupation with relationships	−0.003	0.0304	−0.063	0.056	0.011	1	0.917
Gender * ASQ relationships as secondary	−0.014	0.0277	−0.068	0.040	0.265	1	0.607
Gender * contamination symptoms	0.035	0.2974	−0.548	0.618	0.014	1	0.907

*SE, Standard error; SES, Sexual Excitation Scale; CI, Confidence Interval; OCI-R, Obsessive Compulsive Inventory-Revised; ASQ, Attachment Style Questionnaire*.

**Table 6:** Generalized linear model of SIS1 scores on gender, contamination symptoms and ASQ scores (*n* = 72).

**Outcome: SIS1**	**β**	***SE***	**95% CI**				
			**Lower limit**	**Upper limit**	**Wald *χ^2^***	***df***	***p-*value**
Intercept	2.230	0.9183	0.430	4.030	5.896	1	0.015
Gender	2.073	1.5604	−0.985	5.132	1.766	1	0.184
Age	0.004	0.0063	−0.008	0.017	0.498	1	0.480
Contamination symptoms	−0.176	0.1882	−0.545	0.193	0.871	1	0.351
ASQ confidence	0.010	0.0163	−0.022	0.042	0.365	1	0.546
ASQ discomfort with closeness	−0.013	0.0150	−0.043	0.016	0.775	1	0.379
ASQ relationships as secondary	−0.010	0.0146	−0.038	0.019	0.455	1	0.500
ASQ need for approval	−0.017	0.0157	−0.048	0.013	1.221	1	0.269
ASQ preoccupation with relationships	0.029	0.0157	−0.002	0.059	3.290	1	0.070
Gender * ASQ confidence	−0.028	0.0271	−0.081	0.025	1.092	1	0.296
Gender * ASQ discomfort with closeness	0.005	0.0252	−0.045	0.054	0.036	1	0.850
Gender * ASQ need for approval	−0.009	0.0221	−0.052	0.034	0.161	1	0.688
Gender * ASQ preoccupation with relationships	−0.049	0.0324	−0.113	0.014	2.311	1	0.128
Gender * ASQ relationships as secondary	−0.012	0.0295	−0.069	0.046	0.155	1	0.694
Gender * contamination symptoms	0.690	0.3171	0.069	1.312	4.737	1	0.030

*SE, Standard error; SIS1, Sexual Inhibition Type 1 Scale; CI, Confidence Interval; OCI-R, Obsessive Compulsive Inventory-Revised; ASQ, Attachment Style Questionnaire*.

For SI due to performance consequences (Table 7), women reported lower SI due to performance consequences than men (β = 3.527, *p* = 0.010). In addition, patients with higher ASQ *Confidence* (β = 0.044, *p* = 0.002), higher ASQ *Need for Approval* (β = 0.036, *p* = 0.010) reported lower SI due to performance consequences. Higher contamination symptoms were associated with higher SI due to performance consequences (β = −0.520, *p* = 0.002). There were interaction effects between gender and ASQ *Confidence* and between gender and ASQ *Preoccupations with Relationships* scores*:* women with higher levels on these attachment styles reported higher SI due to performance consequences. There was an interaction effect between gender and ASQ *Relationships as Secondary* scores: women with higher scores on this ASQ subscale had lower SI due to performance consequences (β = 0.059, *p* = 0.023).

**Table 7:** Generalized linear model of SIS2 scores on gender, contamination symptoms and ASQ scores (*n* = 72).

**Outcome: SIS2**	**β**	***SE***	**95% CI**				
			**Lower limit**	**Upper limit**	**Wald *χ^2^***	***df***	***p-*value**
Intercept	0.237	0.8051	−1.341	1.815	0.087	1	0.768
Gender	3.527	1.3681	0.845	6.208	6.645	1	0.010
Age	0.003	0.0056	−0.008	0.014	0.250	1	0.617
Contamination symptoms	−0.520	0.1650	−0.844	−0.197	9.943	1	0.002
ASQ confidence	0.044	0.0143	0.017	0.072	9.721	1	0.002
ASQ discomfort with closeness	−0.012	0.0132	−0.038	0.013	0.879	1	0.349
ASQ relationships as secondary	0.005	0.0128	−0.020	0.030	0.144	1	0.704
ASQ need for approval	0.036	0.0138	0.009	0.063	6.698	1	0.010
ASQ preoccupation with relationships	0.006	0.0138	−0.021	0.033	0.195	1	0.659
Gender * ASQ confidence	−0.075	0.0237	−0.122	−0.029	10.022	1	0.002
Gender * ASQ discomfort with closeness	−0.022	0.0221	−0.065	0.022	0.966	1	0.326
Gender * ASQ need for approval	−0.011	0.0193	−0.049	0.027	0.345	1	0.557
Gender * ASQ preoccupation with relationships	−0.104	0.0284	−0.160	−0.049	13.459	1	0.000
Gender * ASQ relationships as secondary	0.059	0.0259	0.008	0.109	5.157	1	0.023
Gender * contamination symptoms	0.963	0.2780	0.418	1.508	11.996	1	0.001

*SE, Standard error; SIS2, Sexual Inhibition Type 2 Scale; CI, Confidence Interval; OCI-R, Obsessive Compulsive Inventory-Revised; ASQ, Attachment Style Questionnaire*.

Finally, there was an interaction effect between gender and contamination symptoms: women with contamination symptoms had lower SI due to performance consequences (β = 0.963, *p* = 0.001).

### Comparisons on Sexual Arousal Between Patients With and Without Antidepressants

In order to examine whether those OCD patients who reported sexual arousal impairment were more likely to be on concurrent antidepressant medications, a series of Student's *t*-tests were performed. No significant differences in SIS/SES scores emerged between patients who were on antidepressants and those who were not. The scores between the two groups were not significantly different on the SES [*t*_(70)_ = −0.77, *p* = 0.44], on the SIS1 [*t*_(70)_ = −1.13, *p* = 0.26], and on the SES2 [*t*_(70)_ = −1–03, *p* = 0.30].

## Discussion

Obsessive–compulsive disorder is a mental condition that is associated with significant impairment in different quality of life domains (2). One aspect that causes notable distress, but is underestimated and neglected by clinicians, is the impairment in sexual life. Little is known about the processes and factors related to impaired sexual arousal among this clinical population. This is the first study which investigated the role of attachment styles and contamination symptoms as moderators of the relationship between gender and sexual arousal processes amongst OCD patients according to the DCM.

### Hypothesis A: Women Report Lower SE and Higher SI Than Men

A first analysis of our findings detected no significant differences between men and women on SE and SI. This result is in contrast with our hypothesis and extensive literature underlining significant gender differences in sexual behavior, with women showing a lower tendency to SE and a higher propensity toward SI than men (14).

Further analyses based on generalized linear models showed that, in contrast with the hypothesis, women had higher SE than men. Again, in contrast with the hypothesis, gender was not related to SI due to performance failure, but it was related to SI due to performance consequences. Specifically, women reported lower SI due to performance consequences when the effects of contamination symptoms were included in the model.

The fact that women had higher SE and lower SI due to performance consequences than men is unexpected because previous reviews and empirical studies found that female gender is related to a worse quality of life in various interpersonal/intimate domains (6) and to a higher tendency to SI (14–21). In addition, women with OCD were found to have more frequently sexual dysfunctions than men in previous studies [e.g., (4)]. An explanation of the result in the present study might be that men with OCD tend to have a more severe clinical picture than women including a higher number of comorbidities, an earlier onset of OCD symptoms, a more chronic course of the disorder, and greater social impairment and more severe obsessions in some areas related to sexual life such as aggressive/sexual impulses (68). Indeed, the present results showed also that higher general OCD severity was related to lower SE, in accordance with literature data indicating that higher general severity of symptoms is associated with a lower quality of life and functioning in various domains (5, 69). It may be believed that the presence of more intense/distressing obsessions and more frequent/prolonged rituals would distract the individual from the contact with the present moment during sexual encounters and exposure to erotic stimuli (e.g., sexual fantasies). A patient with more severe general symptoms might be only focused on the reduction of negative emotions and might not be used to experience or recognize positive emotions.

Another potential explanation why women had higher SE than men might be related to gender differences in the attitudes toward mental health and help-seeking behaviors, with men generally having a delayed request of a psychiatric help which might compromise their quality of life in different life domains over time, then increase the risk of a more chronic course (70). However, due to the small sample size, we did not have enough power to explore the interaction effects between such clinical features such as general severity or the chronic course and gender. Therefore, these explanations remain speculative. However, the fact that women reported lower levels of SI due to performance consequences than men might be considered in line with the high levels of SI found as indicative of erectile dysfunction in community samples of men (12).

### Hypothesis B: Attachment Anxiety/Avoidance Are Related to Lower SE and Higher SI

The results showed an association between higher confidence in self and others and higher discomfort with intimacy and higher SE. On the one hand, the association between higher confidence and higher SE is in line with our hypothesis and with literature data in community samples showing that secure individuals engage in healthy sexual relationships (22, 26–29). A higher level of SE might help the patient to focus her/his attention on the present moment, and it might be a motivational process driving OCD patients to seek intimacy as a way to satisfy their needs of attachment or reduce negative emotions (71).

On the other hand, the relation between higher discomfort with intimacy and higher SE is in contrast with our hypothesis and with literature data in community samples showing that in individuals with attachment insecurities constant fear of separation and attachment anxiety can lead to a reduction in the need for sexual pleasure (30–37). This unexpected result might be explained by the fact that the unpleasant emotions experienced toward intimacy and the tendency to avoid intimate relations might create a sort of rebound effect with an increase in SE due to the avoidance of sexual encounters and fantasies (72).

In line with our hypothesis, patients with higher confidence reported lower sexual inhibition due to threat of performance consequences thus supporting the notion that a secure attachment style might be a protective factor against sexual impairment. This outcome was confirmed also in the model considering the effects of contaminations symptoms. Indeed, this result was also in line with literature data reported in community samples indicating that individuals with a secure attachment have healthy sexual relationships (22, 26–29). Finally, patients with higher need for approval had lower SI due to performance consequences, in disagreement with the hypothesis. It might be speculated that attachment anxiety based upon approval seeking is characterized by the constant reliance on partner to fulfill safety needs. In the general population, this specific relationship pattern determines the use of intimacy as a mean to satisfy their desire for closeness (73), and this psychological mechanism might explain why patients with higher need for approval were paradoxically less inhibited.

### Hypothesis C: For Women Higher Attachment Anxiety and for Men, Higher Attachment Avoidance Is Related to Lower SE and Higher SI

Female patients with higher confidence in self and others reported lower SE. This result was in contrast with our hypothesis and perhaps it suggests that a secure attachment style might be a protective factor of sexual life among men with OCD but not women. However, in line with our hypothesis, we also found that women with higher discomfort with intimacy reported lower SE. This finding appears consistent with previous research showing significant correlations between both attachment anxiety and attachment avoidance and poor sexual functioning (36, 74, 75). We might speculate that female patients with discomfort with intimacy would be more likely to avoid sexual stimuli and interactions, and this might decrease their levels of desire and interest in sexual encounters, that in turn might be related to a lower SE.

In addition, in line with our hypothesis, women with higher preoccupations for relationships reported higher SI due to performance consequences. This result seems to be in line with literature data in community samples suggesting that women would be more likely to have attachment anxiety than men, and this might compromise their sexual and relationship functioning (35–39). This might suggest that female OCD patients would experience an obsessive focus of their attention on the possibility of losing their partners and/or the need for appearing perfect in their partners' eyes, particularly during sexual interactions. This, in turn, might compromise their ability of freeing themselves to sexual emotions and sensations during sexual encounters, therefore determining sexual inhibition (30).

In addition, in contrast with our hypothesis, female patients with higher confidence had higher SI due to performance consequences. This was an unexpected and paradoxical result. An explanation might be that performance consequences such as the risk of getting contaminated during the sexual act become more important for women with OCD if they have less attachment insecurities since these women are less preoccupied with losing their partners, but they might be more preoccupied with symptoms of OCD during the sexual act. So, attachment insecurities might involve preoccupations regarding the relationship with the partner that paradoxically might distract the woman from the fear of getting contaminated during sexual encounters. However, we did not measure the level of importance perceived by the participants about the relationships and about the risk of getting contaminated; thus, this explanation needs for further support.

Finally, female patients with higher levels of confidence and preoccupations with relationships reported higher SI due to performance consequences, while women considering relationships as secondary had lower levels of SI due to performance consequences.

### Hypothesis D: For Women, Contamination Symptoms Are Related to Lower SE and Higher SI

In line with the majority of the literature data from recent systematic reviews (49–51), women were more likely to report contamination symptoms than men.

In contrast with our expectations, contamination symptoms were not related to SE and they did not interact with gender. An explanation for this result might be that the level of SE is not closely related to physical contact *per se* but to broader aspects of sexual life such as sexual fantasies. Therefore, the presence of significant contamination symptoms might not have a relevant direct effect on this domain of sexual response.

In line with our hypothesis, women with contamination symptoms reported higher SI due to threat of performance failure. Despite these results are preliminary, they suggest that perhaps the presence of contamination symptoms in women might be associated with a stronger SI. Specifically, the presence of significant contamination symptoms in women might reinforce their preoccupation about a failure during the sexual act. Thus, considering this specific type of obsessions and compulsions seems to be useful to better understand the role of gender in sexual inhibition processes. Overall, the moderator role of contamination symptoms for women is in line with literature data in clinical samples showing that this subtype would be more specific to women (56–58) and that it would be a strong predictor of a worse quality of life (76).

In line with our hypothesis, contamination symptoms were associated with higher SI due to performance consequences thus suggesting that the fear for getting contaminated might be associated with a higher propensity for SI due to the possibility of getting contaminated with a sexually transmitted disease during the sexual act. Unexpectedly, women with contamination symptoms had lower levels of SI due to performance consequences. This result was in contrast with our hypothesis. We might speculate that perhaps the higher levels of social avoidance including intimate relationships, commonly observed in female patients with contamination symptoms (77), might lead them paradoxically to consider performance consequences of sexual encounters as less distressing than female patients with other symptoms but with less interpersonal avoidance. However, since we did not control for the effects of avoidance in our model, this explanation remains speculative and needs for further support.

### Comparison Between Patients With and Without Antidepressants

We found no difference in the levels of SE and SI between patients who were on antidepressants and those who were not. This finding is in contrast with most of the literature data (6), indicating that this type of medication can significantly alter sexual functioning. The inclusion of patients on antidepressants should be considered a limitation of the study. Indeed, it may be speculated that this absence of differences might be related to some methodological aspects including the cross-sectional design, the relatively low statistical power, and the lack of control of the effects of potentially confounding variables associated with medications such as the duration of medication intake, dosages, and types of antidepressants.

### Implications for Personalized Treatment OCD

The findings of the present pilot study suggest that the assessment of attachment styles and contamination symptoms should be integrated in a personalized management of OCD patients, particularly for those patients with sexual arousal impairment. Specific psychotherapeutic modules may be added to the standard psychotherapeutic treatment based upon exposure and response prevention (ERP) and/or cognitive restructuring. For example, schema therapy has been found to be a promising strategy to be delivered in combination with ERP for OCD (78). The aim of this type of psychotherapeutic approach is challenging the early maladaptive schemas developed through early adverse relational experiences during childhood, when one or more of basic psychological needs are not satisfied. Taking into account the present findings, we might hypothesize that the introduction of a treatment approach such as schema therapy aimed to target the attachment insecurities might be useful for those OCD patients with sexual impairment. In addition, the present findings point out the need for considering sexual life as a therapy target for OCD patients with contamination symptoms. For this subgroup of OCD patients, a personalized approach might include additional therapeutic ingredients, such as couple or sexual therapy modules.

### Limitations and Future Directions

The cross-sectional design of the study did not allow us to draw firm conclusions about the relation between gender and sexual response. In addition, the moderator roles of attachment styles and contamination symptoms need to be supported by a longitudinal design. Despite attachment styles develop during childhood and adolescence, they may continue to be influenced by adult experiences. Contamination symptoms may wax and wane over time. Thus, both these clinical features might be affected by impaired sexual life. Future research should use longitudinal designs to explore prospectively the role of attachment styles and contamination symptoms on the development of sexual arousal impairment. In addition, the present data were collected in a period before the current pandemic situation. It would be interesting to explore whether sexual life among OCD patients has changed during the pandemic period since contamination symptoms in OCD have been found to be a risk factor for a relapse during the current pandemic (79, 80).

Another limitation concerns the imbalanced men to women ratio and the fact that an a-priori power analysis was not carried out to identify the number of men and women requested to detect significant differences and interaction effects. Due to the small sample size, it was not possible to explore the role of gender on sexual arousal separately in subgroups with other obsessions than contamination ones. It would be worth investigating whether the effect of gender is moderated by other obsession types such as those associated with sexual, moral, or religious themes.

Another interesting point might be to assess whether the role of gender on sexual arousal processes is in turn associated with other relevant outcomes such as couple satisfaction or the presence of sexual dysfunctions. Future studies using mediational analyses should test whether the processes considered by the Dual Control Model can mediate the relation between gender and broader outcomes of sexual life such as couple satisfaction or sexual dysfunctions. Finally, the cross-sectional design and the lack of an investigation on other related factors (i.e., dosages, types of medications) did not allow us to clarify the reasons why there were no differences between patients with and those without antidepressant medication on sexual arousal. Future longitudinal studies based on random assignment to medication should clarify this point. Moreover, the effect of additional clinical features that were not controlled for in our study should be examined, such as anxious and depressive symptoms, as they are very often associated with OCD and they might impact on sexual arousal negatively. It might be interesting to compare sexual arousal across different OCD spectrum conditions such as also skin picking disorder which typically focuses on the body (81).

## Conclusions

The results of the present study show in a clinical group of OCD patients that the relation between gender and sexual arousal processes might be moderated by attachment styles and contamination symptoms. Women with higher discomfort with intimacy but also with higher confidence in self and others would have lower SE, while women with higher preoccupations with relationships but also with higher confidence would be more threatened by potentially negative performance consequences such as getting contaminated with a sexually transmitted disease. Finally, women with contamination symptoms would have higher SI due to threat of performance failure but lower SI due to performance consequences than men.

In conclusion, the present preliminary findings suggest that sexual arousal should be more carefully evaluated during the assessment in clinical practice with OCD patients, and that gender-based effects of attachment styles and contamination symptoms should be taken into account during personalized treatment planning.

## Data Availability Statement

The raw data supporting the conclusions of this article will be made available by the authors, without undue reservation.

## Ethics Statement

The studies involving human participants were reviewed and approved by University of Florence. The patients/participants provided their written informed consent to participate in this study.

## Author Contributions

DD designed the study, conducted the literature searches, wrote the first draft of the paper, and revised the final version. NA collected the data, conducted the literature searchers, and wrote the first draft of the paper. DM designed the study, analyzed the data, and revised the final draft of the paper. FM conducted the literature searches and revised the final draft of the paper. DP collected the data and revised the final draft of the paper. AP designed the study, collected and analyzed the data, and wrote the first draft of the paper. All authors contributed to the article and approved the submitted version.

## Conflict of Interest

The authors declare that the research was conducted in the absence of any commercial or financial relationships that could be construed as a potential conflict of interest.
